# Safety and Optimal Neuroprotection of neu2000 in acute Ischemic stroke with reCanalization: study protocol for a randomized, double-blinded, placebo-controlled, phase-II trial

**DOI:** 10.1186/s13063-018-2746-9

**Published:** 2018-07-13

**Authors:** Ji Man Hong, Mun Hee Choi, Sung-Il Sohn, Yang-Ha Hwang, Seong Hwan Ahn, Yeong-Bae Lee, Dong-Ick Shin, Ángel Chamorro, Dennis W. Choi, on the behalf of the SONIC investigators, Ji Man Hong, Sung-Il Sohn, Yang-Ha Hwang, Seong Hwan Ahn, Yeong-Bae Lee, Dong-Ick Shin, Dennis W. Choi, Eung Yeop Kim, Jin Soo Lee, Jin Wook Choi, Dong Hoon Shin, Min-Ju Yeo, Jaehyuk Kwak, Sung Eun Lee, Jeong-Ho Hong, Sangkil Lee, Yoon-Joo Lee, Min-Joo Lee

**Affiliations:** 1 Department of Neurology, Ajou University School of Medicine, Ajou University Medical Center, 164, World cup-ro, Yongtong-gu, Suwon-si, Kyunggi-do 442-749 Republic of Korea; 2 Department of Neurology, Dongsan Medical Center, Keimyung University, Daegu, Republic of Korea; 3 Department of Neurology, Kyungpook National University Hospital, Daegu, Republic of Korea; 4 Department of Neurology, College of Medicine, Chosun University, Gwangju, Republic of Korea; 5 Department of Neurology, Gachon University Gil Medical Center, Incheon, Republic of Korea; 6 Department of Neurology, Chungbuk National University Hospital, Chungbuk National University College of Medicine, Cheongju, Republic of Korea; 7 Comprehensive Stroke Center, Department of Neuroscience, Hospital Clinic, University of Barcelona and August Pi I Sunyer Biomedical Research Institute (IDIBAPS), Barcelona, Spain; 8 Department of Neurology, Stony Brook University, Stony Brook, NY USA

**Keywords:** Endovascular recanalization, Ischemia and reperfusion, Neuroprotectants, Collateral

## Abstract

**Background:**

The potential of neuroprotective agents should be revisited in the era of endovascular thrombectomy (EVT) for acute large-artery occlusion because their preclinical effects have been optimized for ischemia and reperfusion injury. Neu2000, a derivative of sulfasalazine, is a multi-target neuroprotectant. It selectively blocks *N*-methyl-D-aspartate receptors and scavenges for free radicals. This trial aimed to determine whether neuroprotectant administration before EVT is safe and leads to a more favorable outcome.

**Methods:**

This trial is a phase-II, multicenter, three-arm, randomized, double-blinded, placebo-controlled, blinded-endpoint drug trial that enrolled participants aged ≥ 19 years undergoing an EVT attempt less than 8 h from symptom onset, with baseline National Institutes of Health Stroke Scale (NIHSS) score ≥ 8, Alberta Stroke Program Early CT score ≥ 6, evidence of large-artery occlusion, and at least moderate collaterals on computed tomography angiography. EVT-attempted patients are randomized into control, low-dose (2.75 g), and high-dose (5.25 g) Neu2000KWL over 5 days. Seventy participants per group are enrolled for 90% power, assuming that the treatment group has a 28.4% higher proportion of participants with functional independence than the placebo group. The primary outcome, based on intention-to-treat criteria is the improvement of modified Rankin Scale (mRS) scores at 3 months using a dichotomized model. Safety outcomes include symptomatic intracranial hemorrhage within 5 days. Secondary outcomes are distributional change of mRS, mean differences in NIHSS score, proportion of NIHSS score 0–2, and Barthel Index > 90 at 1 and 4 weeks, and 3 months.

**Discussion:**

The trial results may provide information on new therapeutic options as multi-target neuroprotection might mitigate reperfusion injury in patients with acute ischemic stroke before EVT.

**Trial registration:**

ClinicalTrials.gov, ID: NCT02831088. Registered on 13 July 2016.

**Electronic supplementary material:**

The online version of this article (10.1186/s13063-018-2746-9) contains supplementary material, which is available to authorized users.

## Background

Recent clinical trials of endovascular thrombectomy (EVT) have shown a remarkably positive effect on the functional outcome of patients with acute ischemic stroke presenting with proximal anterior circulation occlusion [1]. However, a considerable number of individuals with stroke still remain disabled despite a higher rate of reperfusion and a striking improvement in clinical outcome by the recent use of stent retrievers [2]. Numerous endeavors have been devoted to attenuating neuronal cell death after ischemic injury, and “excitotoxicity” is considered one of the therapeutic targets for neuroprotection in acute ischemic stroke [3]. Herein, *N*-methyl-D-aspartate (NMDA) receptors play a role in excitatory neurotransmission, and their overactivation results in neuronal death after acute ischemic stroke. NMDA-receptor antagonists prevent such excitatory action of glutamate and cellular calcium overload. Unfortunately, no randomized controlled trials have succeeded in demonstrating the clinical efficacy of such drugs [4]. Potential reasons for the failure are explained by modeling mismatch between preclinical experiments and human stroke, adverse effects of the drugs, inappropriate time window, or complexity of the ischemic cascade [5]. Such limitations are being resolved with improvements in therapeutic strategies. First, the potential of neuroprotective agents as a promising treatment strategy in patients with acute ischemic stroke is being revisited in the EVT era because their preclinical efficacy has been optimized in ischemia and reperfusion models [6]. In this regard, neuroprotective actions of drugs may be optimized when cerebral blood flow is restored in actual clinical practice for acute ischemic stroke. Second, to address the complexity of the ischemic cascade, a multi-target neuroprotective modality, such as therapeutic hypothermia, is gaining evidence in cardiac arrest [7]. Such multi-step or multi-target treatment would be highly beneficial after recanalization in patients with acute ischemic stroke [8].

**Fig. 1:**
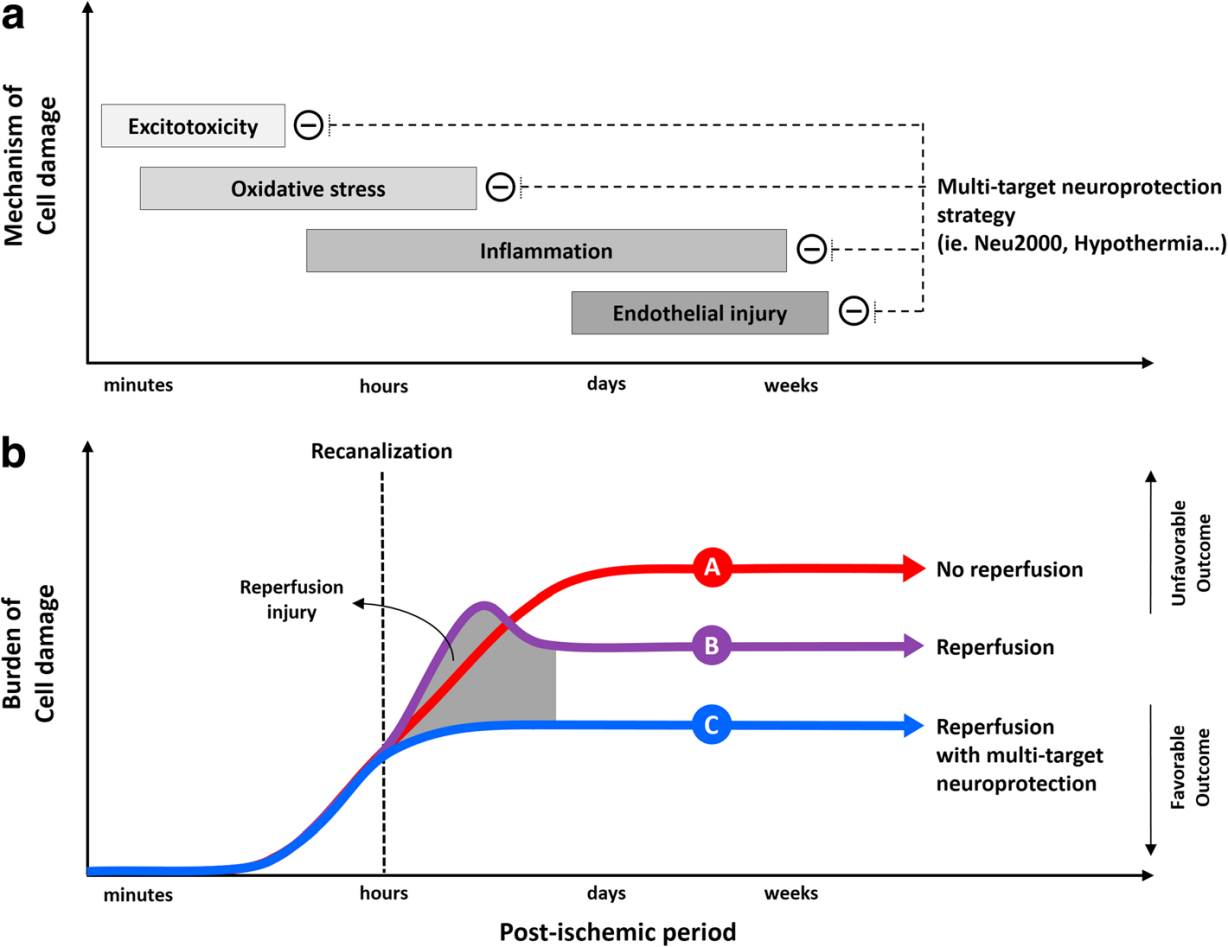
Simplified illustration of possible mechanisms after Neu2000 administration to mitigate reperfusion injury in the setting of recanalization treatment. **a** Ischemic cascade after stroke and proposed effect of multi-target neuroprotection. **b** The expected effect of reperfusion with multi-target neuroprotection on burden of cell damage and functional outcome

Neu2000 (a derivative of sulfasalazine) is a novel, multi-target neuroprotectant that combines modest (micromolar) NR2B subtype-selective blockade of NMDA receptors with potent (nanomolar) scavenging of reactive oxygen species [9, 10] (Fig. 1). It has been suggested that the prior failure of more potent, and subtype-unselective NMDA-receptor antagonist drugs in stroke trials may have been partly due to excessive reduction of intracellular free calcium levels and consequent enhancement of ischemic neuronal apoptosis [11]. Neu2000 seeks to blunt acute excitotoxicity without risking this downside, and then to additionally target downstream free-radical damage, which is prominently triggered by vascular reperfusion. The neuroprotective potential of Neu2000 has been well demonstrated in preclinical animal stroke models with a favorable efficacy and a wide range of therapeutic window profile [9, 10, 12, 13]. In human phase-I studies, there are no serious adverse events including psychosis [14]. The current trial aims to provide proof-of-concept for use of Neu2000 as an adjunct neuroprotective agent together with state-of-the-art EVT in patients presenting to stroke centers with acute ischemic stroke.

## Methods

### Design

**Fig. 2:**
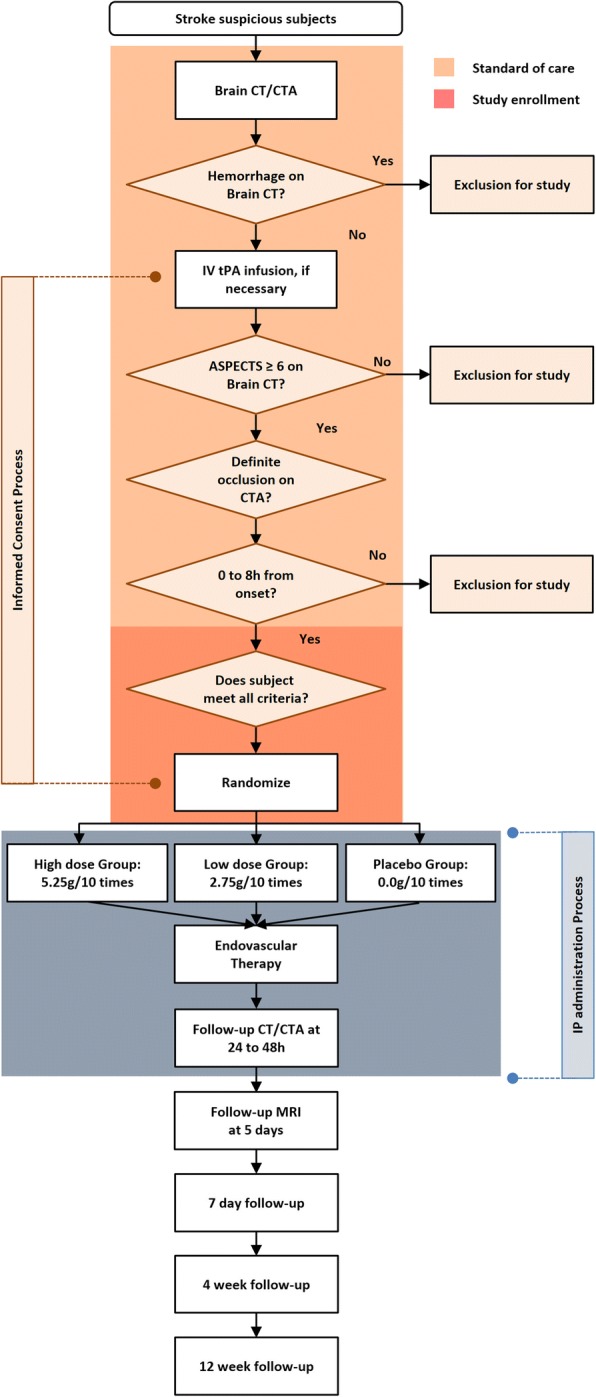
The flowchart of SONIC trial. *CT* computed tomography, *CTA* computed tomography angiography, *IV* intravenous, *tPA* tissue plasminogen activator, *ASPECTS* Alberta Stroke Program Early CT Score, *IP* investigational product, *MRI* magnetic resonance imaging

The Safety and Optimal Neuroprotection of neu2000 in acute Ischemic stroke with reCanalization (SONIC) trial is a multicenter, national, prospective, randomized, three-arm, parallel-group, double-blinded, placebo-controlled, phase-II clinical study with blinded-endpoint evaluation carried out in the Republic of Korea. This study aims to examine the effects of neuroprotectants in EVT-attempted patients within 8 h of stroke onset. All participating centers had received Institutional Review Board approval prior to trial initiation. Emergency medical consent is obtained from caregivers before study enrollment. Approval was obtained from the Institutional Review Board of each center. The flowchart of this study is shown in Fig. 2. The Standard Protocol Items Recommendations for Interventional Trials (SPIRIT) checklist is attached as Additional file 1.

### Patient population

We will enroll acute ischemic stroke patients with moderate to severe neurological deficits (at least National Institutes of Health Stroke Scale (NIHSS) score ≥ 8) and imaging confirmation of proximal vessel occlusion in the anterior circulation. Before randomization, local investigators will assess imaging criteria with the Alberta Stroke Program Early CT Score (ASPECTS), definite proximal artery occlusion, and collateral status on baseline computed tomography (CT) and CT angiography (Fig. 2). Detailed study inclusion and exclusion criteria are shown in Table 1.

**Table 1:** Study inclusion and exclusion criteria

Inclusion criteria	
1. Age ≥ 19 years	
2. Subject who is presented to hospitals after onset of brain ischemic symptoms from the base of last normal state and can start endovascular therapy in accordance with standard practice guidelines within 8 h after the symptom onset	
3. NIHSS ≥8 at screening time point	
4. Subject whose activity is possible without the help of others in the general condition 1 day before the ischemic stroke onset and prestroke Barthel Index scores > 90	
5. Subject whose cerebral CT and CTA imaging confirm acute ischemic stroke and symptomatic intracranial occlusion at screening and whose occlusion site considered the cause of acute ischemic stroke meets the following conditions: T- or L-type ICA occlusion, MCA M1 occlusion, MCA-M1 equivalent (2 or more MCA-M2, anterior temporal artery is not regarded M2)	
6. Subject with ASPECTS ≥ 6 on cerebral non-contrast CT	
7. Subject who spontaneously submit a written informed consent to participate in this trial	
Clinical exclusion criteria	
1. A medical history of hypersensitivity against aspirin (salicylates), sulfasalazine, or 5-ASA	
2. Subject whose heart diseases were confirmed at screening: Subject who was diagnosed with myocardial infarction within 6 months; subject who had severe arrhythmia evoking clinical symptoms (respiratory difficulties, tachycardia, etc.) within 6 months; subject whose ECG measured at the emergency room confirms the following results: A. pulse rate < 50 or > 120 beats/min. B. 2nd or 3rd degree AV block. C. congenital or acquired QT syndrome. D. ventricular pre-excitation syndrome	
3. Subject who was diagnosed with heart failure ≥ New York Heart Association class II Class I: patients with no limitation of activities; they suffer no symptoms from ordinary activities Class II: patients with slight, mild limitation of activity; they are comfortable with rest or with mild exertion Class III: patients with marked limitation of activity; they are comfortable only at rest Class IV: patients who should be at complete rest, confined to bed or chair; any physical activity brings on discomfort and symptoms occur at rest	
4. Subject who has a contraindication to iodinated contrast media	
5. Subject who is receiving renal replacement therapy such as dialysis due to acute or chronic renal failure	
6. Subject who is diagnosed with cancer or received cancer therapy within 6 months or has recurrent or metastatic cancer	
7. Subject who shows a high body temperature of 38 °C or more or who need antibiotic therapy due to infectious diseases	
8. Subject who takes pharmacotherapy for liver diseases such as hepatitis or liver cirrhosis	
9. Subject who is pregnant or lactating. In case of women of child-bearing potential, only subjects who are confirmed as not being pregnant can participate in this trial	
10. Subject who participated in other clinical studies within the past 3 months. In case of participation in an observational study without medication, the subject can participate in this trial	
11. Subject who was determined inappropriate for participation in this trial due to other reasons	
Imaging exclusion criteria	
1. Baseline CT evidence of intracranial hemorrhage 2. Baseline CT evidence of intracranial tumor on presentation 3. Baseline CTA shows that the site of occlusion considered to be the cause of acute ischemic stroke meets the following conditions: A. MCA + PCA or MCA + ACA occlusion in carotid T/L-type B. Occlusion of a bilateral intracranial large artery C. Simultaneous involvement of anterior and posterior circulation 4. Absence of the collateral circulation corresponding to one of the followings: CTA imaging shows absence or minimal collateral circulation at ≤ 50% of MCA territories, compared with pial filling of the contralateral side	

*NIHSS* National Institutes of Health Stroke Scale, *CT* computed tomography, *CTA* computed tomography angiography, *ICA* internal carotid artery, *MCA* middle cerebral artery, *ASPECTS* Alberta Stroke Program Early CT Score, *ASA* aminosalicylate, *ECG* electrocardiography, *PCA* posterior cerebral artery, *ACA* anterior cerebral artery

### Randomization

Participants will be randomly assigned in a 1:1:1 fashion to one of three arms: (1) high-dose, (2) low-dose, or (3) placebo. Randomization is based on computer-generated cards before study initiation and will be handed to authorized personnel in a sealed envelope. All study investigators and participants are masked to treatment allocation except investigational product (IP)-preparing personnel. The randomization schedule is not accessible except for safety reasons, until completion of this trial. Study drugs and the placebo are provided in an identical package.

### Treatment

A study subject is enrolled after clinical judgment with inclusion and exclusion criteria on the basis of acute-onset neurological manifestation, confirmed by imaging for arterial vessel occlusion. Written informed consent is obtained from the patient or a legal representative before enrollment. After enrollment, the study subject undergoes rapid endovascular recanalization therapy. The neuro-interventionist uses any available modality (stent retriever or aspiration thrombectomy) or its combination to achieve safe recanalization. In addition, the use of a balloon-guided catheter is recommended in the relevant internal carotid artery. All groups will receive the best current standard of care as described in the current local guidelines for acute stroke management: This represents the fact that they will receive tissue plasminogen activator intravenously in a 4.5-h window if they meet the accepted criteria [15].

The assigned groups are injected with intravenous administration of IP as a high-dose, low-dose, or placebo of Neu2000KWL (2-hydroxy-5-(2,3,5,6-tetrafluoro-4-trifluoromethyl-benzylamino)-benzoic acid) before recanalization attempt by the personnel who are blinded to treatment allocation. The first infusion is intended to be initiated before thrombus retrieval. Subsequent injection will be administered twice per day (12-h interval) during five consecutive days. In the high-dose group, the initial infusion dose is 750 mg mixed with 250 mL of saline; the subsequent nine doses are 500 mg (total dose: 5250 mg). In the low-dose group, the initial infusion dose is 500 mg, and the subsequent nine doses are 250 mg (total dose: 2750 mg). The placebo group receives 250 mL of saline 10 times. The infusion volume is 250 mL in all groups. All patients receive care in the stroke unit or neuro intensive care unit. Follow-up imaging studies will be obtained as computed tomography (CT) angiography at 24–48 h from the first infusion and subsequent magnetic resonance imaging within 24 h from the last infusion. Follow-up images play a role in the detection of adverse effects of the study drug.

**Fig. 3:**
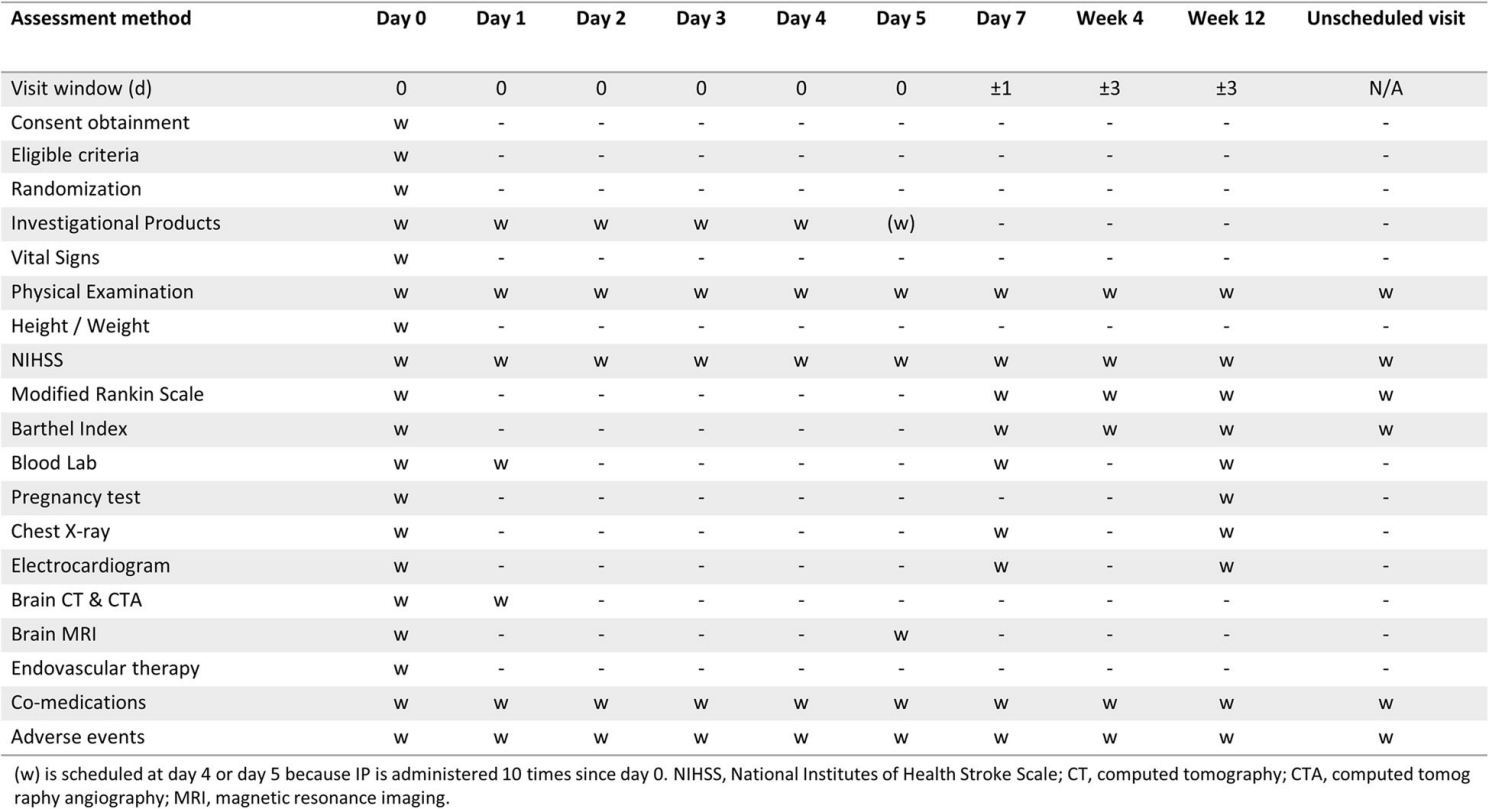
Schedule of enrollment, interventions, and assessments in SONIC trial as per Standard Protocol Items: Recommendations for Interventional trials (SPIRIT)

All patients visit at the fourth week and third month after randomization. During follow-up, modified Rankin Scale (mRS) score, NIHSS score, and Barthel Index (BI) will be determined. Adverse events are collected throughout the study. Detailed follow-up schedules and procedures are shown in Fig. 3.

### Blinding

Treatment assignment will remain blind in both the patient and treating physician except IP-preparing personnel. The IP-preparing personnel are not involved in any other aspect of the study. Study outcomes at baseline, day 7, the fourth week, and third month will be assessed with standardized forms and procedures by separate certified investigators blinded to the treatment. Finally, the Blinded-endpoint Monitoring Board will determine the primary endpoint through central adjudication. Results of neuroimaging will also be assessed by the imaging core laboratory team who are unaware of the treatment allocation.

### Study outcome

The primary outcome will be assessed by a dichotomous model: the proportion of patients with mRS 0–2 and that with mRS 3–6 at 3 months. Safety outcomes are all serious adverse events and symptomatic intracranial hemorrhage with neurologic deterioration (NIHSS score ≥ 4) on cerebral CT within 24–48 h after randomization [16]. The secondary outcomes are as follows: distributional change of mRS, difference in NIHSS score, ratio of NIHSS score 0–2, and ratio of BI ≥ 90 at 1, 4 weeks, and 3 months. All local principal investigators (PI) have a consensus meeting for imaging interpretation before the start of the trial and present regular meeting bi-annually.

### Imaging endpoints

After completion of patient enrollment, all imaging data will be evaluated by the core imaging laboratory. Stroke neurologists, neuroradiologists, and neurointerventionists with expertise in acute stroke management will perform core laboratory imaging analyses to ensure consistent grading and eliminate possible bias after de-identification and blinding of clinical data. The core laboratory team will assess the ASPECTS, infarct volume, recanalization defined by modified Thrombolysis in Cerebral Infarction grades, and the presence of hemorrhagic transformation. All core laboratory readings will be performed independently by two experienced readers. Discrepancies will be resolved by a third, independent reader.

### Sample size estimates

The primary hypothesis of this study is that patients receiving Neu2000 before EVT will have an increased proportion of 3-month functional independence (mRS 0–2), compared with those receiving placebo. The expected proportions of functional independence are 41.6% and 70.0% in the placebo and Neu2000 groups, respectively. Such assumptions are based on the results of CT-based EVT trials (MR CLEAN [17] and ESCAPE [18]), and the preclinical Neu2000 research with transient occlusion model [9]. We chose the target sample size of 63 participants per group to provide 90% power, at a two-sided alpha of 0.05 to prove a treatment effect. The total sample size was determined to be 210 considering a 10% dropout rate for the primary endpoint.

### Statistical analyses

The primary outcome analysis is based on the intention-to-treat principle. Statistical testing for the primary endpoint is conducted using the chi-square test for the comparison of functional independence among the three arms at a two-sided significance threshold of 10%. In case of significant differences, comparisons between the placebo and low-dose Neu2000 groups, and placebo and high-dose Neu2000 groups will be conducted. For secondary outcome analyses, the Cochran-Mantel-Haenszel test, the two-way analysis of variance test, and the chi-square test will be performed on the per-protocol population. In addition, multivariate analysis will be used to adjust for potential confounders, such as age, stroke severity, time to EVT onset, previous stroke, atrial fibrillation, diabetes mellitus, ASPECTS, and T-type internal carotid artery occlusion. If there are missing data despite best efforts to keep all missing data to a minimum, then missing outcome data will be not substituted for the primary outcome analysis.

### Study organization and data monitoring body

The Steering Committee consists of local principal investigators, stroke neurologists, neuroradiologists, and neurointerventionists from each center, members of the Advisory Committee, and independent trial statisticians. The Trial Executive Committee consists of six principal investigators. Subcommittees exist for blinded-endpoint assessment, adverse event adjudication, and imaging analysis. All data will be entered into a web-based trial management system by trained local research nurses. The trial coordinator at the central trial office will carefully review and fully check all local data against source data.

## Discussion

The SONIC trial intends to show that Neu2000 before EVT can be safely administered and have a positive effect on tissue damage and clinical outcomes. Despite recent advances in EVT, considerable functional dependence (14–58%) still remains in patients with successful recanalization (60–90%) [2]. Ameliorating reperfusion injury is a possible target for new drug development; new drug trials are ongoing with uric acid, therapeutic hypothermia, and various neuroprotectants in EVT-attempted patients with stroke [3]. In addition, imaging findings according to the assigned groups will be informative in an effort to ameliorate reperfusion injury including edema and hemorrhagic transformation.

Our trial also intends to show that Neu2000 before EVT can be safely administered and have a positive effect on tissue damage and clinical outcomes in patients with acute ischemic stroke. This proof-of-concept trial will also show whether it would be more effective in specific clinical targets. In addition, this trial will investigate whether the escalating dose mitigates the ischemic damages shown in preclinical experiments [9]. Single-target neuroprotectants have failed in acute stroke clinical trials despite remarkable success in preclinical studies [19]. Multi-target neuroprotective strategy using Neu2000 is challengeable in its activity on timely blocking the sequential ischemic cascade, which is responsible for eventual neuroglial death after ischemic stroke (Fig. 1).

This trial is a novel study focusing on adjuvant neurovascular protection against ischemia-reperfusion injury in humans by selecting eligible patients as described above. Neu2000 is a first-in-class multi-target drug inhibiting NMDA-receptor-induced excitotoxicity, free-radical toxicity, and blood-brain barrier disruption. Future phase-III trials with solid evidence, like shift analysis, should be considered for establishing the neuroprotective properties in acute ischemic stroke.

## Trial status

This report describes the protocol, version 4.0, 19 June 2017. SONIC is currently recruiting study participants. The first patient was randomized in September 2016, and the targeted end date for recruitment is December 2018.

### The SONIC investigators

*Trial principal investigator*

Ji Man Hong, Ajou University School of Medicine, Ajou University Medical Center.

*Local principal investigators*

Ji Man Hong, Ajou University School of Medicine, Ajou University Medical Center; Sung-Il Sohn, Dongsan Medical Center; Yang-Ha Hwang, Kyungpook National University Hospital; Seong Hwan Ahn, Chosun University; Yeong-Bae Lee, Gachon University Gil Medical Center; Dong-Ick Shin, Chungbuk National University Hospital.

*Advisory Committee*

Dennis W. Choi, Stony Brook University; Ángel Chammorro, University of Barcelona, and August Pi I, Sunyer Biomedical Research Institute (IDIBAPS).

*Imaging core laboratory*

Eung Yeop Kim, Gachon University Gil Medical Center; Jin Soo Lee, Jin Wook Choi, Ajou University School of Medicine, Ajou University Medical Center.

*Blinded-endpoint Assessment Committee*

Dong Hoon Shin, Gachon University Gil Medical Center; Min-Ju Yeo, Chungbuk National University Hospital; Jaehyuk Kwak, Dongsan Medical Center.

*Safety Review Committee*

Sung Eun Lee, Ajou University School of Medicine, Ajou University Medical Center; Jeong-Ho Hong, Dongsan Medical Center; Sangkil Lee, Chungbuk National University Hospital.

*Independent trial statisticians*

Yoon-Joo Lee, Min-Joo Lee, Medical excellence.

## Additional file

Additional file 1: Standard Protocol Items: Recommendations for Interventional Trials (SPIRIT) 2013 Checklist: recommended items to address in a clinical trial protocol and related documents*. Additional file 1: of Safety and Optimal Neuroprotection of neu2000 in acute Ischemic stroke with reCanalization: study protocol for a randomized, double-blinded, placebo-controlled, phase-II trial. (DOCX 50 kb)

## Abbreviations

ASPECTS: Alberta Stroke Program Early CT score
BI: Barthel Index
EVT: Endovascular thrombectomy
IP: Investigational product
mRS: Modified Rankin Scale
NIHSS: National Institutes of Health Stroke Scale
NMDA: *N*-methyl-D-aspartate
SONIC: Safety and Optimal Neuroprotection of neu2000 in acute Ischemic stroke with reCanalization
